# The coastal ocean response to the global warming acceleration and hiatus

**DOI:** 10.1038/srep16630

**Published:** 2015-11-16

**Authors:** Enhui Liao, Wenfang Lu, Xiao-Hai Yan, Yuwu Jiang, Autumn Kidwell

**Affiliations:** 1 State Key Laboratory of Marine Environmental Science, College of Ocean and Earth Sciences, Xiamen University, Xiamen 361005, China; 2 Center for Remote Sensing, College of Earth, Ocean and Environment, University of Delaware, Newark, DE 19716, USA; 3 Joint Institute for Coastal Research and Management, University of Delaware/Xiamen University, USA/China

## Abstract

Coastlines are fundamental to humans for habitation, commerce, and natural resources. Many coastal ecosystem disasters, caused by extreme sea surface temperature (SST), were reported when the global climate shifted from global warming to global surface warming hiatus after 1998. The task of understanding the coastal SST variations within the global context is an urgent matter. Our study on the global coastal SST from 1982 to 2013 revealed a significant cooling trend in the low and mid latitudes (31.4% of the global coastlines) after 1998, while 17.9% of the global coastlines changed from a cooling trend to a warming trend concurrently. The trend reversals in the Northern Pacific and Atlantic coincided with the phase shift of Pacific Decadal Oscillation and North Atlantic Oscillation, respectively. These coastal SST changes are larger than the changes of the global mean and open ocean, resulting in a fast increase of extremely hot/cold days, and thus extremely hot/cold events. Meanwhile, a continuous increase of SST was detected for a considerable portion of coastlines (46.7%) with a strengthened warming along the coastlines in the high northern latitudes. This suggests the warming still continued and strengthened in some regions after 1998, but with a weaker pattern in the low and mid latitudes.

Despite the continued increase of atmospheric greenhouse gases, the global surface mean temperature has remained flat since 1998^1^,^2^. The global climate exhibited a shift from a rapid global surface warming to an unexpected deceleration in the global surface warming with a notable cooling in the eastern tropical Pacific Ocean (a La-Niña-like pattern) and a strengthening of the trade winds^1^,^3^,^4^. This change in the acceleration of the global surface warming has received much notice with the title of “global surface warming hiatus”^1^,^2^,^3^,^4^. To be consistent with previous studies, we refer to this time period (after 1998) as the hiatus period. Due to heterogeneously distributed aerosols and changes in the atmospheric/oceanic circulation, the regional ocean responses (warming/cooling) are not uniform^5^,^6^. Recent studies reported that the main locations of the warming hiatus are in the central and eastern Pacific Ocean and over the northern continents, especially Eurasia. This indicates that there is a regionality to the impact of the global climate shift^4^,^7^,^8^. Lima and Wethey^9^ reported a high warming rate for 71.6% of the global coastlines during the last three decades (1982–2010). However, there is limited information on the variations of warming/cooling for the global coastlines during the hiatus period. Since strong scientific interest has focused on the mechanism of the global surface warming hiatus^1^,^10^,^11^,^12^ and few studies have paid attention to the coastal effect, it is imperative to study the corresponding coastal effects of the global surface warming hiatus.

Approximately 50% of the world’s population lives within 200 kilometers of coast and nearly half of the ocean’s ecosystem goods and services come from coastal marine ecosystems^13^,^14^. This vital region is highly influenced by a multitude of forcing mechanisms (i.e. anthropogenic processes, sediment transport, ocean circulation, wind stress, and large scale climate variability)^15^. Recently, extreme sea surface temperatures (SST) occurred in many coastal areas and caused serious physiological stress and large casualties to various coastal ecosystems^16^,^17^,^18^,^19^,^20^,^21^,^22^,^23^. These extreme SSTs (i.e., extremely cold and hot events) were proven to be difficult for temperature-sensitive coastal coral reef ecosystems, some of the most diverse and productive communities on the earth^24^. For instance, an extremely hot event was observed in the Caribbean Sea (2005), and extremely cold events were witnessed in the Taiwan Strait (the China coast, 2008) and the Florida Keys (the eastern U.S. coast, 2010)^16^,^17^,^18^,^19^. Additionally, the extreme SSTs can also cause disastrous impacts on non-coral-reef ecosystems (e.g., the western coast of Australia in 2011 and the northeastern U.S. coast in 2012)^20^,^23^ and rocky-reef communities (e.g., the Mediterranean in 2003)^22^. Therefore, the understanding and prediction of the effects of the global surface warming hiatus on the coast are beneficial to the scientific field, policy makers, and general population. By studying the variability of coastal SST, extremely hot days (EHDs), and extremely cold days (ECDs) in a global context, this study details how global surface warming hiatus translates into regional patterns.

## Results

### Time series of yearly SST

**Figure 1:**
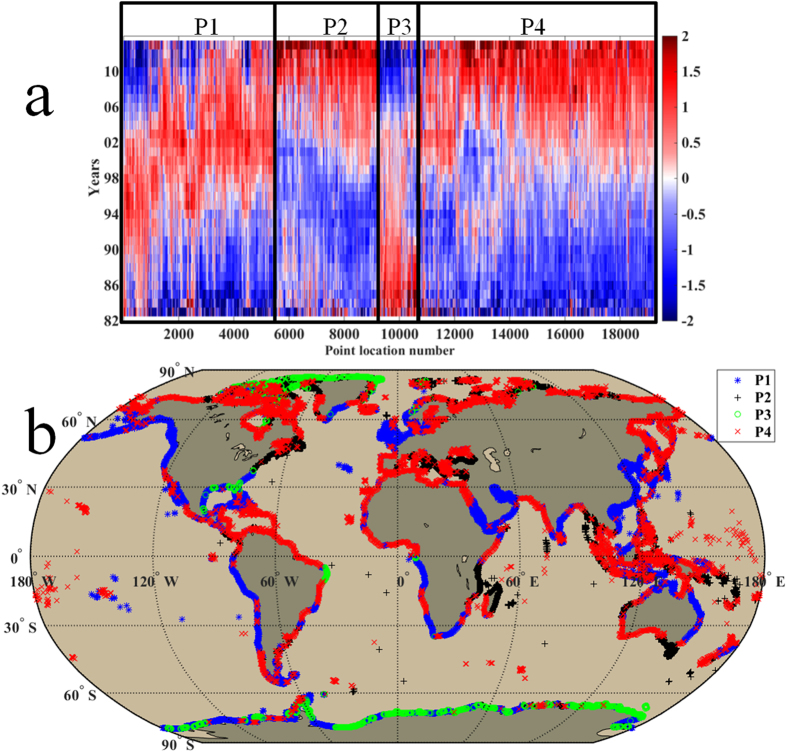
The time series of standardized yearly SST and four changing patterns. (**a**) The time series of standardized yearly SST at each coastal location (total 19276), sorted by four changing patterns. (**b**) The distribution of four changing patterns along the world’s coastlines. The first pattern is that the SST increased in the warming period (1982–1997), then decreased in the hiatus period (1998–2013), which is P1 in the panel a and blue star mark in the panel b. The second pattern is SST decreased in the first period, then increased in the second period, which is P2 in the panel a and black plus mark in the panel b. The third pattern is SST continued decreasing, which is P3 in the panel a and green circle mark in the panel b. The fourth pattern is SST continued increasing, which is P4 in the panel a and red x mark in the panel b. See Method section for the methods to obtain the yearly SST time series. We generated the two sub-panels (**a,b**) using Matlab and integrated the sub-panels into this figure.

The SST data is separated into two different periods (1982–1997, hereafter called warming period; 1998–2013, hereafter called hiatus period) for analysis. Globally, the trend of global mean coastal SST was 0.17 ± 0.11 °C/decade (95% confidence interval) in the warming period and reduced to 0.11 ± 0.09 °C/decade during the hiatus period. In order to better depict variations in these two time periods, yearly SSTs are filtered using a 10-year moving average (Fig. 1). Specifically, temporal variations in these two periods can be classified into four patterns (Fig. 1) that are (a) SST increased in the warming period (0.26 ± 0.10 °C/decade, mean trend), and then decreased in the hiatus period (−0.24 ± 0.11 °C/decade), which is P1 in the Fig. 1a and blue star mark in the Fig. 1b; (b) SST first decreased (−0.11 ± 0.08 °C/decade), and then increased (0.27 ± 0.09 °C/decade), which is P2 in the Fig. 1a and black plus mark in the Fig. 1b; (c) SST continued decreasing (−0.07 ± 0.06 and −0.09 ± 0.11 °C/decade), which is P3 in the Fig. 1a and green circle mark in the Fig. 1b; (d) SST continued increasing (0.20 ± 0.11 and 0.31 ± 0.11 °C/decade), which is P4 in the Fig. 1a and red x mark in the Fig. 1b. The first pattern occupied 31.4% of the global coastlines (Fig. 1b) and primarily distributed in the low and mid latitudes (60°S–60°N, defined in the Method section). This pattern changing from warming to cooling is compatible with the present global climate shift. The second pattern is discontinuously distributed in 17.9% of the global coastlines (e.g., the western tropical Pacific and Indian Ocean, Fig. 1b). The third pattern primarily appeared in the Polar Regions. The fourth pattern (continued warming), corresponding with the global warming, is located in nearly half of the global coastlines (46.7%, Fig. 1b). These findings suggest that, while coastal warming was still the major pattern during the hiatus period, cooling was observed along numerous coastlines (35.4%), with a majority of the cooling coastlines occurring in the low and mid latitudes.

### Global patterns

**Figure 2:**
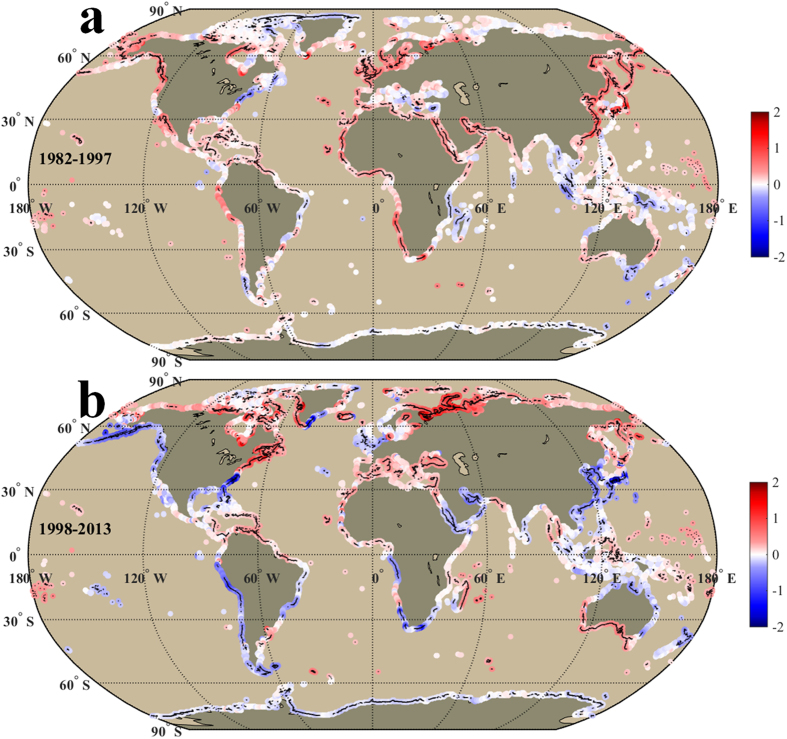
The linear SST trends (unit: °C/decade) along the world’s coastlines in the warming (1982–1997, **a**) and hiatus periods (1998–2013, **b**). Black points/lines in the shading color indicate the trends in those locations are significant in statistics (P < 0.05). We generated the two sub-panels (**a,b**) using Matlab and integrated the sub-panels into this figure.

The patterns of the global coastal SST trends (Fig. 2) demonstrate a notable warming in 68.78% of the coastlines in the low and mid latitudes in the warming period. In contrast, during the hiatus period, a significant coastal cooling was detected in nearly half (48.33%) of the coastlines in the low and mid latitudes, while a significant warming occurred in 82.42% of the coastlines in the high northern latitudes (60°N–90°N). Compared with warming period, the area ratio of global coastal warming in the hiatus period reduced from 73.19% to 64.60%.

**Figure 3:**
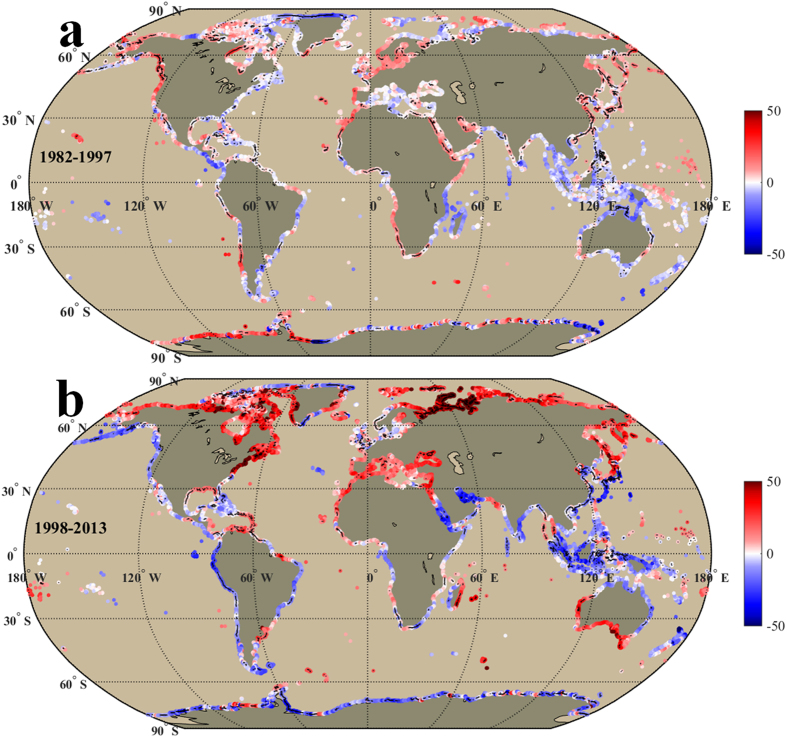
Same as Fig. 2, but shows the linear trend (days/decade) of annual frequency of extremely hot days in the warming (1982–1997, **a**) and hiatus periods (1998–2013, **b**). Black points/lines in the shading color indicate the trends in those locations are significant in statistics (P < 0.05). We generated the two sub-panels (**a,b**) using Matlab and integrated the sub-panels into this figure.

**Figure 4:**
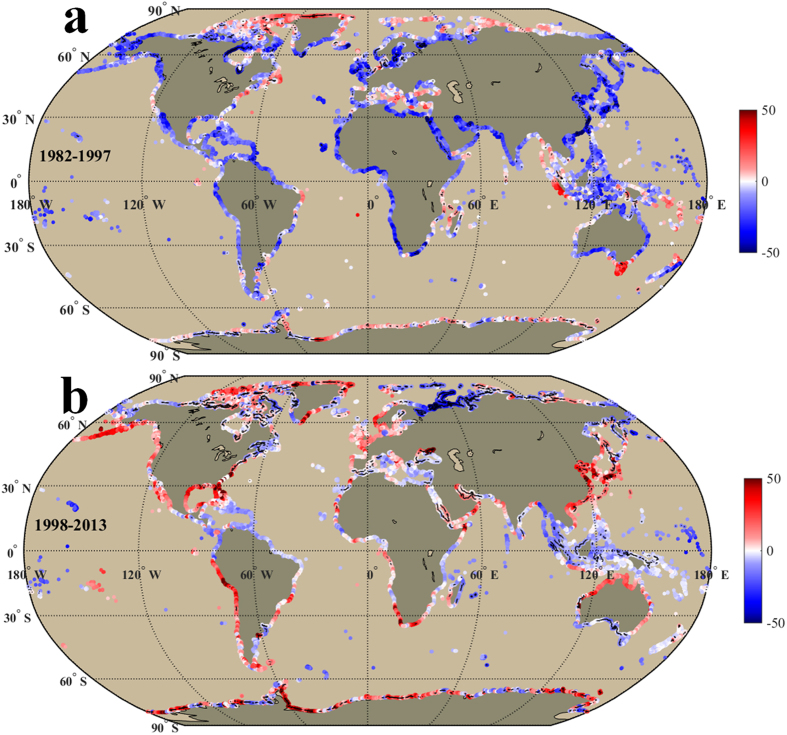
Same as Fig. 2, but shows the linear trend (days/decade) of annual frequency of extremely cold days in the warming (1982–1997, **a**) and hiatus periods (1998–2013, **b**). Black points/lines in the shading color indicate the trends in those locations are significant in statistics (P < 0.05). We generated the two sub-panels (**a,b**) using Matlab and integrated the sub-panels into this figure.

Figs 3 and 4 exhibit linear trends of annual frequency of EHDs and ECDs, which are positively and negatively correlated with SST trends, respectively. The correlation coefficients between SST trends and trends of EHDs and ECDs were 0.53 (EHDs) and −0.77 (ECDs) in the warming period and 0.73 (EHDs) and −0.62 (ECDs) in the hiatus period respectively (*P* < 0.01). Therefore, the region with higher warming (cooling) rate tends to have more EHDs (ECDs), suggesting a rising probability of occurrence of extremely hot (cold) event. However, the moderate correlations (0.53–0.77) indicate the trends of EHDs and ECDs along some coastlines may be influenced by other factors (e.g., SST variance) in addition to the SST trends. The positive trends of EHDs occupied almost half of the global coastlines with similar area ratios (51.8% and 51.9%) in these two periods, but different distributions. Compared with the warming period, the positive trends of EHDs in the hiatus period were mainly observed in the high northern latitudes (Fig. 3). The area ratio of ECDs with positive trend in the global coast increased from 23.9% in the warming period to 44.6% in the hiatus period. The increase in the number of ECDs primarily occurred in the low and mid latitudes, notably in the Pacific Ocean (Fig. 4) during the hiatus period. This observation intimates a modified regional pattern of warming and cooling after 1998: a significant cooling along the coast of the low and mid latitudes, principally in the Pacific Ocean, and a notable warming along the coasts of the high northern latitudes even though there was a hiatus in the increase of the global surface mean temperature. As a result, the corresponding numbers of EHDs and ECDs increased rapidly along these warming (high northern latitudes) and cooling (low and mid latitudes) coasts, leading to a higher probability of extremely hot and cold events, respectively.

### Pacific

In the Pacific Ocean, the coasts (excluding the Antarctica region) grew warmer (~0.17 °C/decade, trend of mean SST in this area) in the warming period and colder (~−0.05 °C/decade) in the hiatus period (Fig. 2). The changes in the SST trends in the Pacific Ocean are the largest of all the ocean basins. These variations are in keeping with the present report that the hiatus is associated with the Pacific Ocean^1^,^3^. The overarching distribution of the coastal SST trends (Fig. 2b) in the hiatus period were an eastern coastal cooling and a western coastal warming in the North Pacific (except for the China and Japan), and a coastal cooling in the South Pacific (except for the Maritime Continent and South Pacific Islands). The pattern in the Pacific may be related to the present climate features (the negative PDO phase and eastern tropical Pacific cooling)^1^,^7^. In the western Pacific, the cooling trend along the China and Japan coast (−0.69 ± 0.44 °C/decade), opposing to the overarching distribution, implies other influencing factors (e.g., the East Asian Monsoon)^25^.

The number of EHDs (Fig. 3) in the hiatus period increased significantly for warming coastlines (e.g., the Northwestern Pacific, 21.46 ± 7.90 days/decade). The number of ECDs (Fig. 4) increased along cooling coastlines, particularly for the coastlines of China and Japan (25.30 ± 11.75 days/decade), the Alaskan Peninsula (21.13 ± 19.74 days/decade), and the western coastlines of South America (19.27 ± 11.98 days/decade). The China coast witnessed a distinct reversal, in that the trend of EHDs decreased from 8.33 ± 7.22 to −6.53 ± 5.01 days/decade (Fig. 3) and ECDs increased from −36.57 ± 23.29 to 25.30 ± 11.75 days/decade (Fig. 4). The consequence of the increasing trend of ECDs is a higher probability of an extreme cold event (e.g., the Taiwan Strait cold disaster in 2008^18^). If this trend persists, the frequency and intensity of extreme events may increase, leading to ecological and economic instability for these regions. The Alaskan Peninsula coast is another example of a trend reversal of EHDs and ECDs (see detailed trend values in Supplementary Table S1 online).

### Atlantic

In the Atlantic Ocean (excluding the Antarctic region), during the warming period, coastal warming (~0.16 °C/decade) was the characteristic feature (Fig. 2a). In the hiatus period, although coastal warming (~0.09 °C/decade) was still the dominant feature, coastal cooling was observed in part of the North Atlantic and most of the South Atlantic. During the hiatus period, the North Atlantic pattern of the coastal SST trends (Fig. 2b) was a tri-polar pattern, with a warming SST trend (0.54 ± 0.24 °C/decade) in the north (north of Cape Hatteras and the Labrador Sea), a cooling SST trend (−0.37 ± 0.33 °C/decade) in the middle (south of Cape Hatteras, the southeastern Greenland Island, and the North Sea), and a warming SST trend (~0.15 °C/decade) in the south (the tropical Atlantic). The tri-polar pattern may be associated with the present negative phase of the North Atlantic Oscillation (NAO)^26^,^27^,^28^,^29^. In the South Atlantic, the distribution of coastal warming and cooling areas were discontinuous, indicating different influencing factors in different coastal areas.

The corresponding number of EHDs in the North Atlantic increased significantly in the eastern part of North America (north of Cape Hatteras, 35.41 ± 17.94 days/decade), the Labrador Sea (26.33 ± 16.26 days/decade), the Caribbean Sea (~17.5 days/decade), and the Mediterranean Sea (18.52 ± 10.39 days/decade, Fig. 3). The increase of ECDs was exhibited in the eastern part of North America (south of Cape Hatteras, 27.2 ± 24.7 days/decade), and along most of the South Atlantic coast. The trends of EHDs and ECDs along the eastern North American coast reversed at the Cape Hatteras, where the EHDs increased from −3.19 ± 4.19 to 35.41 ± 17.94 days/decade to the north of Cape Hatteras (Fig. 3), and the ECDs increased from −11.4 ± 14.1 to 27.2 ± 24.7 days/decade to the south of Cape Hatteras during the hiatus period (Fig. 4). Coinciding with the increase of EHDs and ECDs, an extremely hot (cold) event occurred to the north (south) Cape Hatteras during the hiatus period^19^,^21^,^23^. In the Caribbean Sea, the trend of EHDs increased from ~−0.2 to ~17.5 days/decade, inducing a rising probability of extremely hot events (e.g., the 2005 coral reef bleaching event in the Caribbean Sea^16^). In the South Atlantic, the largest increase of EHDs appeared along the Argentina coast (20.4 ± 7.4 days/decade) and the largest increase of ECDs occurred along the western coast of South Africa (29.6 ± 14.6 days/decade).

### Indian Ocean

In the Indian Ocean, the coastlines (excluding the Antarctic region) revealed reverse SST trends between the warming and hiatus periods (Fig. 2). In the warming period, coastal warming rate was high (~0.14 °C/decade) in the Northern Indian Ocean, and low (~0.01 °C/decade) in the South Indian Ocean. However, in the hiatus period, this pattern was reversed. The coastal warming rate was low (negative, ~−0.03 °C/decade) in the North Indian Ocean and high (~0.07 °C/decade) in the South Indian Ocean. Specifically, in the hiatus period, the most significant coastal cooling occurred in the Red Sea (~−0.11 °C/decade) and Persian Gulf (−0.36 ± 0.29 °C/decade), while the most significant coastal warming happened along the eastern portion of Madagascar Island (0.33 ± 0.16 °C/decade) and the western and southern part of Australian coast (0.33 ± 0.21 °C/decade). The significant coastal warming in the western part of Australia is likely influenced by the recent swing to the negative phase of the Interdecadal Pacific Oscillation (IPO) and enhanced El Niño Southern Oscillation variance since 1970^30^,^31^. However, in the North Indian Ocean, there may be other factors influencing the coastal SST. Raitsos *et al*.^32^ indicated air temperature is the key parameter that influenced the Red Sea SST, which implied the SST cooling during the hiatus period in the Red Sea may be related to atmospheric forcing.

During the hiatus period, the corresponding number of EHDs increased along the coastlines around the South Indian Ocean, especially for the eastern shore of Madagascar Island (22.8 ± 18.7 days/decade) and the western and southern part of Australian coast (~28 days/decade, Fig. 3). As a result, there is an increased probability of an extremely hot event in these coastal areas (e.g., the 2011 extremely hot event in the western part of Australia^20^). However, the mean number of ECDs in the North Indian Ocean showed a negative trend (~−0.9 days/decade). This reduction in ECDs is inconsistent with the cooling trend (~−0.03 °C/decade). The inconsistencies suggest a decreased SST variance or standard deviation in the hiatus period (see Supplementary Fig. S1a online). However, further studies are needed to explain the decreased SST variance or standard deviation. The significant increase in the number of ECDs appeared along the northern coast of Australia (~17.7 days/decade) where the cooling trend was ~−0.14 °C/decade (Fig. 4).

### Polar Regions

In the Arctic Ocean, SST trends (0.37 ± 0.30 °C/decade) and trends of EHDs (24.8 ± 5.4 days/decade) increased very significantly in the hiatus period, which is consistent with reports of remarkably high temperature trends and ice reduction trends^33^,^34^,^35^. The rapid coastal warming in the Arctic Ocean indicates a significant warming signal in the high northern latitudes. This signifies an extremely hot event might happen if the current trend persists. In the Southern Ocean, the Antarctic coast showed a moderate cooling (−0.08 ± 0.04 °C/decade) which corresponds the recent report that the sea ice expanded slightly over the last decade^36^. The number of ECDs increased (10.1 ± 5.9 days/decade) in the Antarctic coast during the hiatus period.

## Discussion

Lima and Wethey^9^ reported that the last three decades (1982–2010) witnessed a high warming rate in 71.6% of the global coastlines. However, with the same data set, the coastal SST trends illustrate exceptional trend reversals when the last three decades are separated into the warming (1982–1997) and hiatus (1998–2013) periods. In the warming period, most of the global coastlines (68.19%) showed a warming trend consistent with global warming. However, during the hiatus period, the warming trend reversed in many coastlines of the low and mid latitudes (31.4% of the global coastlines). The distribution of these trend reversals matches with multiple reports that the global surface warming hiatus is mainly concentrated in the low and mid latitudes, especially in the Pacific Ocean^1^,^3^.

Additionally, 17.9% of the global coastlines changed from cooling to warming in the hiatus period. These trend reversals, distributed in the low and mid latitudes, may be attributed to natural climate variability instead of anthropogenic-induced warming. There may be two reverse SST variability patterns (i.e., from warming to cooling and from cooling to warming) in response to the global surface warming hiatus. However, further study is needed to distinguish the exact causes of these coastal trends.

Although SST trend reversals are apparent for many coastlines (49.3%, 31.4% + 17.9%), a continuous increase of SST could be detected for a considerable portion of the global coastlines (46.7%). Notably, the high northern latitudes demonstrated an accelerated warming even though in the hiatus period. The continued warming was concentrated in the high northern latitudes, while the effect of global climate hiatus was focused in the low and mid latitudes.

The timing of the warming/cooling trend reversal in the North Pacific and North Atlantic was in conjunction with the recent phase shifts of the PDO and the NAO. This suggests the coastal cooling around the Eastern Pacific and the coastal warming along the coast of the western part of Australia are likely influenced by the recent swing to the negative phase of the PDO^1^,^7^,^25^,^30^,^31^. The tri-polar pattern (warming SST trends in the north and south, and a cooling SST trend in the middle) in the North Atlantic may be associated with the present negative phase of the North Atlantic Oscillation (NAO)^26^,^27^,^28^,^29^. Additionally, other factors (e.g., ocean circulation and monsoons) may play a significant role in the SST variability^37^. For example, the cooling of the China and Japan coasts does not match with the negative phase of the PDO and may be related to the recent strengthening of East Asian Winter Monsoon^25^. Therefore, further study is needed to detail the mechanisms of SST variation for different coastlines.

The corresponding EHDs and ECDs trends have similar variations with the SST trend spatially and temporally. Spatially, the increase of EHDs was observed in the warming coast (e.g., the eastern portion of Madagascar Island, western and southern part of Australia, Caribbean Sea, Mediterranean Sea, eastern part of North America (north of Cape Hatteras), and much of the Arctic Ocean) in the hiatus period (Fig. 3). The number of ECDs increased in the cooling coast (the Eastern Pacific, China and Japan, northern part of Australia, Red sea, Persian Gulf, South Africa, eastern part of North America (north of Cape Hatteras), and some coastlines in both polar regions) in the hiatus period (Fig. 4). Temporally, the trend of global mean EHDs (ECDs) was ~−0.74 (−10.5 ± 8.89) days/decade in the warming period and increased to ~3.96 (~1.65) days/decade in the hiatus period. In contrast to the warming period, the sum of EHDs and ECDs increased globally in the hiatus period (Figs 3 and 4). This means the probability of extreme events (hot or cold) might increase if the global surface warming hiatus continues. The increased sum of EHDs and ECDs may be related to a rising coastal area of large positive and negative SST trends from the warming period to the hiatus period (see Supplementary Fig. S2).

In addition to the SST trend, SST variance may influence the variation of EHDs (ECDs). For example, the mean number of ECDs showed a negative trend (~−0.9 days/decade) when the coast grew colder (~−0.03 °C/decade) in the North Indian Ocean. These inconsistencies along the coastlines around the North Indian Ocean illustrate a decreased standard deviation from the warming period to hiatus period (see Supplementary Fig. S1a online). This suggests the reduced number of ECDs for cooling coastlines is associated with a decreased standard deviation. Due to the same reason, there may be a reduced number of EHDs for coastlines with an increased SST (see Supplementary Fig. S1b online). The decreased standard deviation indicates a less varied SST, which can be shown by the change in the probability density functions between these two periods (see Supplementary Fig. S3 online). If the standard deviation increases, the ECDs (EHDs) might increase even if the SST increases (decreases) along the inconsistent coastlines (see Supplementary Fig. S4 online). The standard deviation mainly decreased in the low latitude and increased in the mid and high latitudes from the warming period to the hiatus period (see Supplementary Fig. S5 online). These coastlines with increased standard deviation are susceptible to extreme hot (cold) events, even if these coastlines are cooling (warming).

The global mean surface air temperature shows an increase of approximately 0.05 ± 0.1 °C/decade from 1998 to 2012, when described by a linear trend^38^. The eastern tropical Pacific Ocean warms by approximately 0.08–0.1 °C/decade from 1900 to 2008, similar in magnitude to the tropical Indian Ocean and central tropical Atlantic Ocean^39^. However, the SST trend was −0.69 ± 0.44 °C/decade along the China Coast, 0.54 ± 0.24 °C/decade along the eastern North American coast, 0.33 ± 0.21 °C/decade along the western coast of Australia in the hiatus period. In contrast to the global mean and open ocean, coastal areas have large and heterogeneous responses, which can have a significant, complicated impact on coastal ecosystems. For example, the temperature-sensitive coral reefs, which normally live above 16 °C, experienced temperatures as low as 11.73 °C during a cold event in the Taiwan Strait (China Coast) in 2008. This event resulted in large casualties to the entire coastal ecosystem^17^. A rise of 0.1 °C in the Caribbean Sea can trigger 35% and 42% increases in the geographic extent and intensity of coral bleaching, respectively^40^. By providing an understanding of the SST variability and its consequences in a global context, this study is significant to understanding spatial pattern of extreme event, designing conservation strategies, and mitigating negative ecosystem responses.

## Methods

### Data

The SST data used in this study is NOAA’s Optimum Interpolation (OI) ¼ Degree Daily SST (also known as Reynolds 0.25 v.2), which is obtained from NOAA’s National Climatic Data Center (http://www.ncdc.noaa.gov/oisst/data-access). There are two SST analysis products developed using OI. The product with only AVHRR infrared satellite SST data is used for long-term time series. A large-scale adjustment of satellite biases with respect to the *in situ* data is included in the data. Many studies of decadal SST variability, based on OISST, indicate there is no evident decadal scale error^41^,^42^. The OISST shows very small biases in comparison with coastal *in-situ* SST data^9^,^43^. The coastal pixel used for analysis is the pixel which is closest to land but with a land contamination of less than 50%^9^.

### The yearly SST

The yearly SST, computed by taking annual mean of daily SST data, is first smoothed by a 10-year moving average, and then is transformed into standardized data (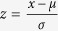, z is the standardized data, x is the smoothed yearly SST data, 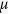 is the mean value, and 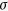 is the variance).

### Trend calculation

The SST trend in the study is calculated as the slope of the linear regression of seasonally detrended daily SST. In order to decrease errors in the SST OI estimates (e.g., random, sampling, and bias error), the slope is computed through a weighted least squares method, whose weights are inversely proportional to the variance of the SST OI (i.e., ∝1/SD^2^). The degrees of freedom were adjusted using the Quenouille procedure to account for temporal autocorrelation^44^.

### Confidence interval

The confidence interval in the study is stated at the 95% confidence level. The standard error of the trend is computed by 
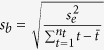
, 
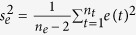
, 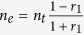, where 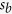
is the standard error, 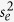 is the variance of the linear regression residuals, *t* is the time index, 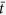 is the average time index, 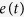 is the linear regression residual, 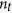 is the total number of time series, 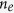 is the adjusted number of time series after removing temporal autocorrelation, 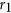 is the lag-1 temporal autocorrelation coefficient of 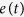
44. In the study, the confidence interval is shown as 0.17 ± 0.11. If the margin of error (“radius”) of a confidence interval is larger than the trend value (e.g., −0.13 ± 0.25), this means the result is not significant. However, the trend can still indicate a decreasing or increasing tendency, and the trend value is shown as ~0.13.

### Annual frequency of extremely cold and hot days

In order to analyze the probability of variations of hot and cold events, the linear trends of annual frequency of extremely hot and cold days are given. The thresholds of extremely low and high temperature are defined as the 5th and 95th percentiles of the standardized anomalies of the raw SST (1982–2013) at each location separately. Then, the annual frequencies of daily anomalies exceeding the threshold values were computed. In the linear regression, the Quenouille procedure is adopted to adjust the degrees of freedom like SST trend calculation. The unit of trend of annual frequency of extremely hot and cold days is the number of days per decade ± trend confidence interval.

### Mean trend

The global (regional) mean warming/cooling rate is computed based on global (regional) mean data (area-weighted average). The area ratio in this study is also based on area-weighted average.

### Standard deviation

At each location, the standard deviation of SST in the warming (hiatus) period is calculated by 
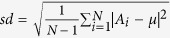
, where *A* is the time series of SST at each location, *N* is the number of variable *A* (SST), 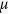 is the mean of *A*.

### Low, mid, and high latitudes

In this study, low latitude is defined as 30°S–30°N, mid latitude is 30°S–60°S and 30°N–60°N, high latitude is 60°S–90°S and 60°N–90°N.

## Additional Information

**How to cite this article**: Liao, E. *et al*. The coastal ocean response to the global warming acceleration and hiatus. *Sci. Rep.* **5**, 16630; doi: 10.1038/srep16630 (2015).

## Supplementary Material

Supplementary Information
