# Supplementary material for: The coastal ocean response to the global warming acceleration and hiatus
Source: Sci Rep. 2015 Nov 16;5:16630. doi: 10.1038/srep16630 (PMC4644973; doi:10.1038/srep16630)
Supplement: Supplementary Information [file srep16630-s1.pdf]

The coastal ocean response to the global warming acceleration and hiatus

Enhui Liao[1,2,3], Wenfang Lu[1,2,3], Xiao-Hai Yan[2,3,*], Yuwu Jiang[1,3], Autumn Kidwell[2]

[1]State Key Laboratory of Marine Environmental Science, and College of Ocean and Earth Sciences, Xiamen University, Xiamen 361005, China

[2]Center for Remote Sensing, College of Earth, Ocean and Environment, University of Delaware, Newark, DE 19716, USA

[3]Joint Institute for Coastal Research and Management, University of Delaware/Xiamen University, USA/China

(*Corresponding author. Tel.: +1-302-831-3694. E-mail: xiaohai@udel.edu)

Supplementary Information contains:

Supplementary Figures S1-S3

Supplementary Table S1

Supplementary Figure S1 The inconsistent points between SST trends and trends of extremely cold (hot) days. **a**, the SST decreased and the extremely cold days decreased; **b**, the SST increased and the extremely hot days decreased. The shading color is the standard deviation difference between warming period and hiatus period. The positive (negative) value means the standard deviation is larger (smaller) in the hiatus period than the warming period. We generated the two sub-panels (a and b) using Matlab and integrated the sub-panels into this figure.

Supplementary Figure S2 The histogram of SST trends for percentage of global coastal area between the warming and hiatus periods. We generated the figure using Matlab.

Supplementary Figure S3 The probability density functions between the warming and hiatus periods in the Red Sea (a) and Arctic Ocean (b). The histogram is normalized with a uniform bin width (0.1 ℃/decade). The line is calculated by probability density function with mean and standard value. We generated the figure using Matlab.

Supplementary Figure S4 Same as Figure S1, the inconsistent points between SST trends and trends of extremely cold (hot), **a**, the SST increased and the extremely cold days increased; **b**, the SST decreased and the extremely hot days increased. We generated the two sub-panels (a and b) using Matlab and integrated the sub-panels into this figure.

Supplementary Figure S5 The standard deviation difference between warming and hiatus periods. The positive (negative) value means the standard deviation is larger (smaller) in the hiatus period than the warming period. We generated the figure using Matlab.

Supplementary Table S1 The summary table for the SST trends, trends of extremely hot days (EHDs) and extremely cold days (ECDs) in the key regions

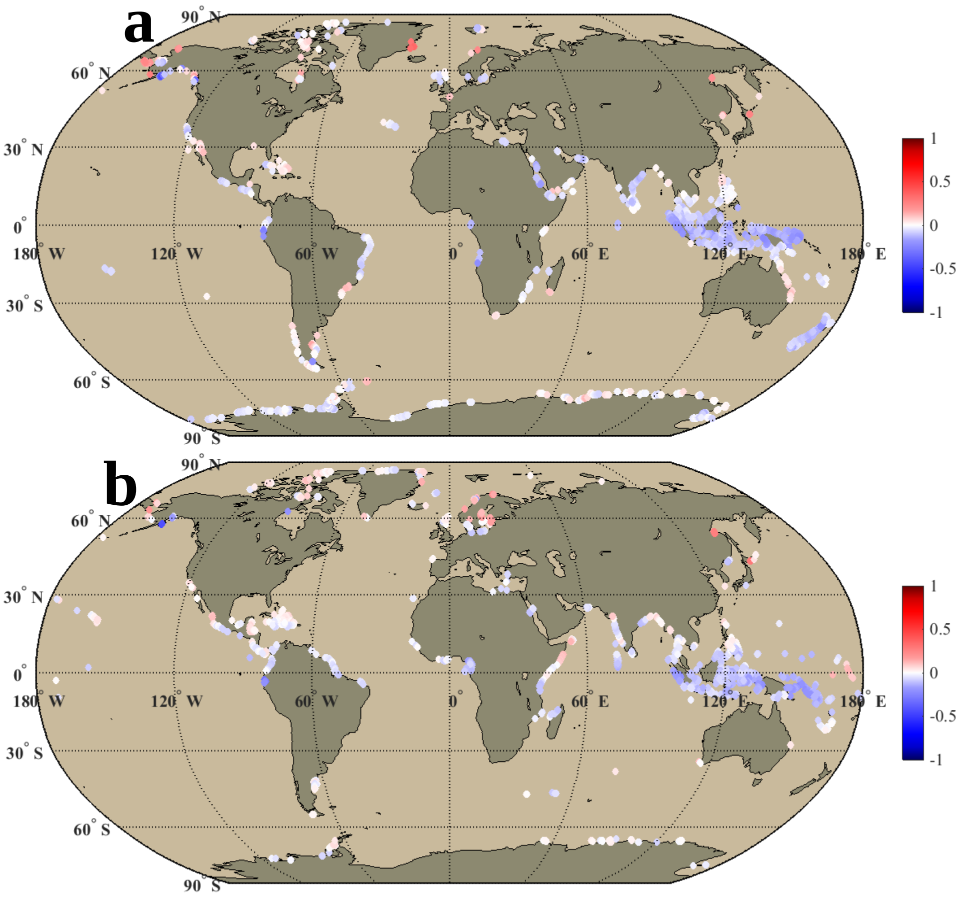

Supplementary Figure S1 The inconsistent points between SST trends and trends of extremely cold (hot) days. **a**, the SST decreased and the extremely cold days decreased; **b**, the SST increased and the extremely hot days decreased. The shading color is the standard deviation difference between warming period and hiatus period. The positive (negative) value means the standard deviation is larger (smaller) in the hiatus period than the warming period. We generated the two sub-panels (a and b) using Matlab and integrated the sub-panels into this figure.

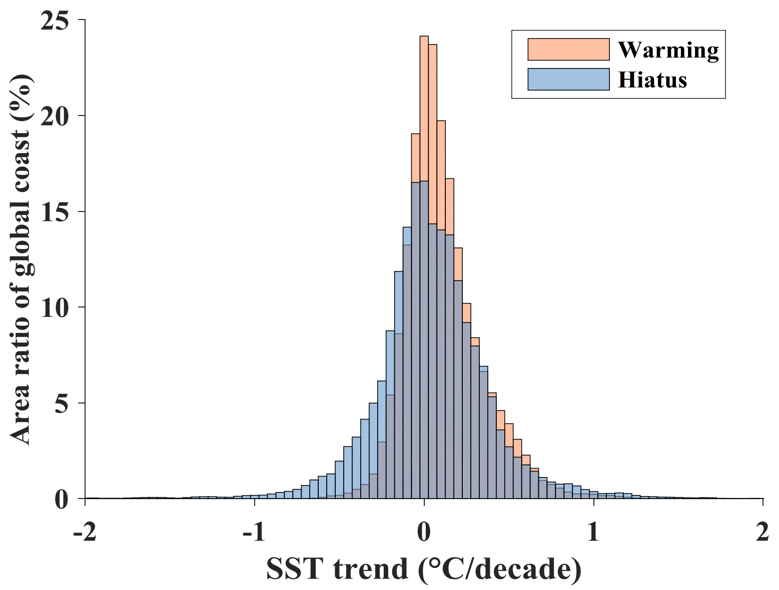

Supplementary Figure S2 The histogram of SST trends for percentage of global coastal area between the warming and hiatus periods. We generated the figure using Matlab.

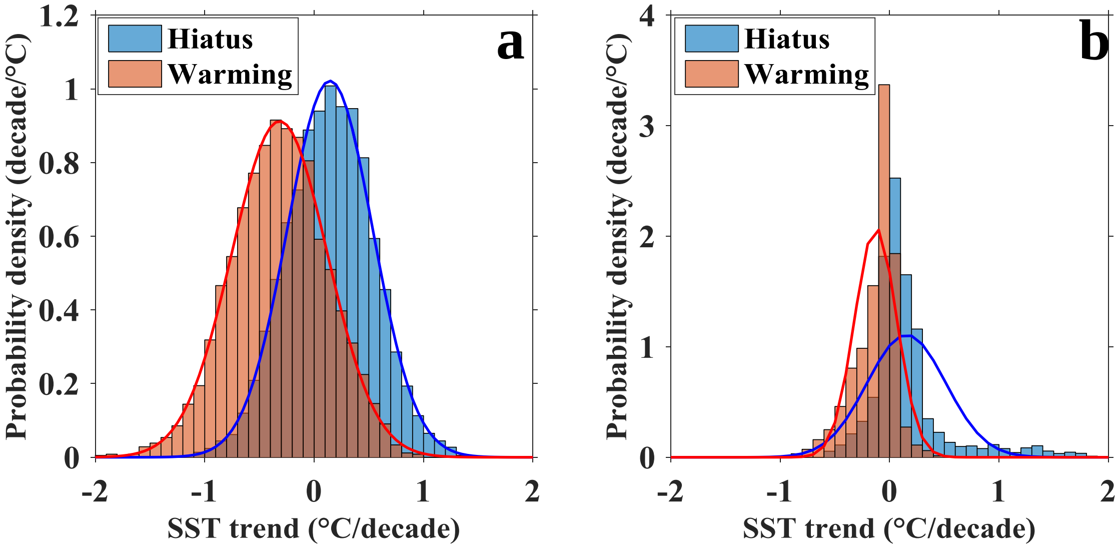

Supplementary Figure S3 The probability density functions between the warming and hiatus periods in the Red Sea (a) and Arctic Ocean (b). The histogram is normalized with a uniform bin width (0.1 ℃/decade). The line is calculated by probability density function with mean and standard value. We generated the figure using Matlab.

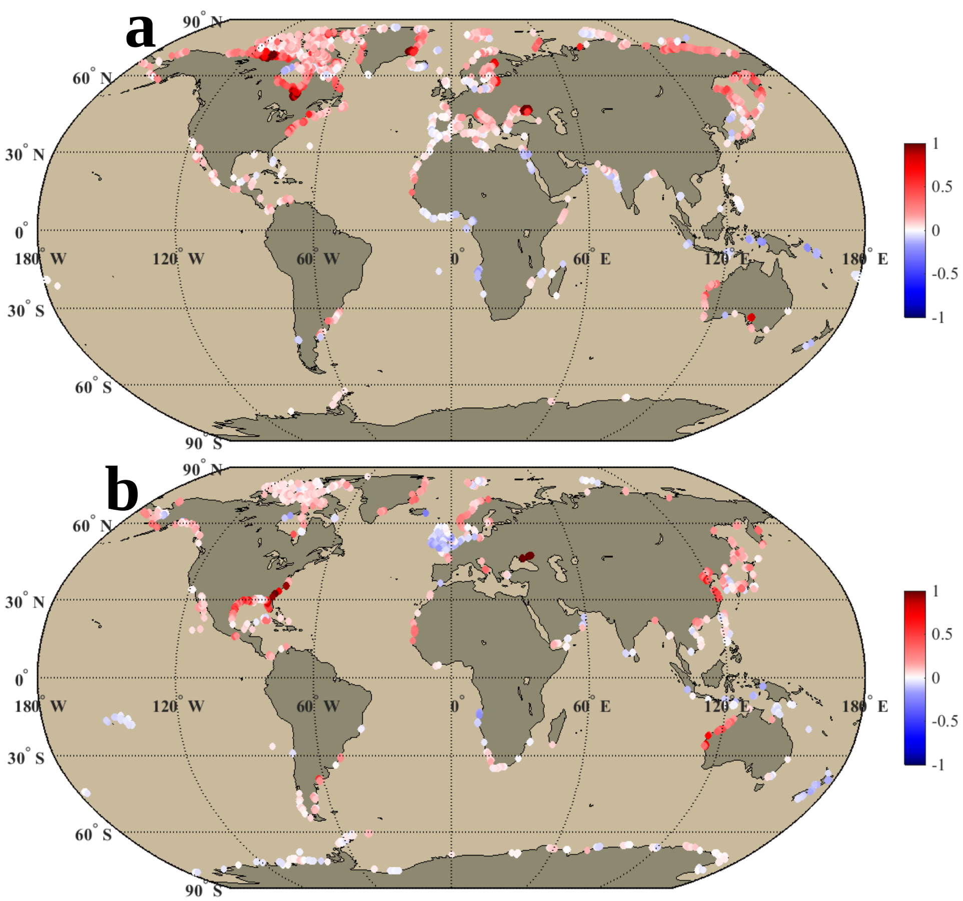

Supplementary Figure S4 Same as Figure S1, the inconsistent points between SST trends and trends of extremely cold (hot), **a**, the SST increased and the extremely cold days increased; **b**, the SST decreased and the extremely hot days increased. We generated the two sub-panels (a and b) using Matlab and integrated the sub-panels into this figure.

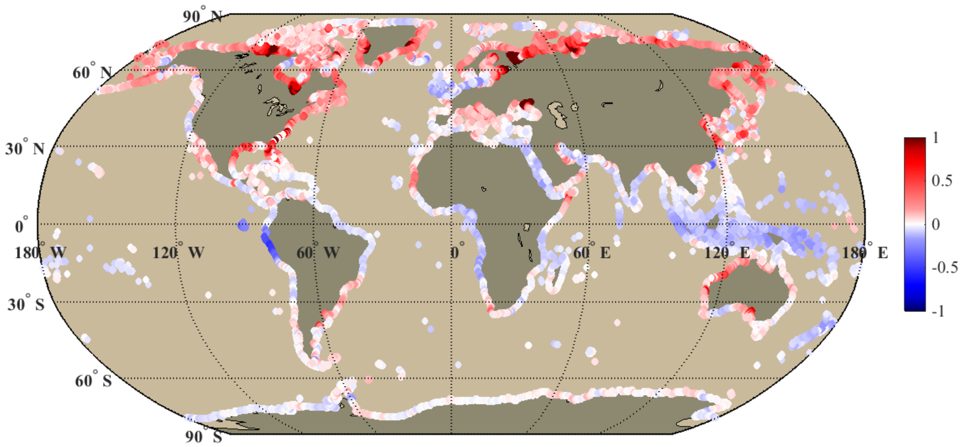

Supplementary Figure S5 The standard deviation difference between warming and hiatus periods. The positive (negative) value means the standard deviation in the hiatus period is larger (smaller) than that in the warming period. We generated the figure using Matlab.

Supplementary Table S1 The summary table for the SST trends, trends of extremely hot days (EHDs) and extremely cold days (ECDs) in the key regions

Region name	SST trend (℃/decade)		EHDs trend (days/decade)		ECDs trends (days/decade)	
	Warming	Hiatus	Warming	Hiatus	Warming	Hiatus
China and Japan coast	0.65±0.27	-0.69±0.44	8.33±7.13	-6.53±5.01	-36.57±23.29	25.30±11.57
Alaskan Peninsula	0.21±0.29	-0.49±0.46	2.64±15.26	-14.88±45.41	-14.71±10.02	21.13±19.74
Western South America	0.18±0.62	-0.36±0.36	4.15±37.33	-11.92±15.66	-11.16±6.81	19.27±11.98
Eastern North America (North of Cape Hatteras)	-0.13±0.25	0.54±0.24	-3.19±4.19	35.41±17.94	0.91±5.08	-3.04±8.74
Eastern North America (South of Cape Hatteras)	0.16±0.47	-0.37±0.33	-2.0±7.71	7.35±9.72	-11.42±14.1	27.2±24.7
Labrador Sea	0.19±0.19	0.38±0.22	3.29±2.66	26.33±16.26	-16.98±21.67	-2.89±5.36
North Sea	0.4±0.53	-0.17±0.46	10.66±4.58	1.45±27.75	-10.49±37.25	10.64±8.83
Caribbean Sea	0.12±0.14	0.24±0.17	-0.19±6.64	11.6±30.21	-17.08±12.52	-8.46±8.41
Mediterranean Sea	0.09±0.24	0.22±0.24	1.67±4.33	18.52±10.39	-6.82±11.44	-0.15±2.78
Argentina coast	0.01±0.36	0.19±0.35	-3.77±14.33	20.4±7.45	-8.10±15.35	1.82±13.12
Western South Africa	0.49±0.26	-0.49±0.25	8.67±12.33	-3.31±12.98	-31.15±41.82	29.6±14.6
Red Sea	0.29±0.14	-0.11±0.14	8.01±7.07	-9.05±13.36	-22.54±17.67	-3.14±5.03
Persian Gulf	0.40±0.35	-0.36±0.29	7.87±11.0	-27.56±20.07	-25.78±34.87	8.64±8.57
Madagascar Island	-0.09±0.16	0.33±0.16	-8.64±10.32	22.79±18.71	-4.74±25.65	-10.82±7.43
Western and southern part of Australia	0.07±0.20	0.33±0.21	-1.97±10.40	28.08±30.46	-12.9±12.9	-2.26±10.11
Arctic Ocean	0.12±0.18	0.38±0.30	2.45±2.34	24.79±5.43	-6.7±6.7	-3.16±7.66
Southern Ocean	0.01±0.03	-0.08±0.04	1.69±8.23	-9.55±11.74	0.34±5.31	10.12±5.78
